# Caspase-1 Dependent IL-1β Secretion Is Critical for Host Defense in a Mouse Model of *Chlamydia pneumoniae* Lung Infection

**DOI:** 10.1371/journal.pone.0021477

**Published:** 2011-06-23

**Authors:** Kenichi Shimada, Timothy R. Crother, Justin Karlin, Shuang Chen, Norika Chiba, V. Krishnan Ramanujan, Laurent Vergnes, David M. Ojcius, Moshe Arditi

**Affiliations:** 1 Division of Pediatrics Infectious Disease and Immunology, Cedars-Sinai Medical Center and David Geffen School of Medicine, University of California at Los Angeles, Los Angeles, California, United States of America; 2 Department of Surgery, Cedars-Sinai Medical Center, Los Angeles, California, United States of America; 3 Department of Human Genetics, University of California Los Angeles, Los Angeles, California, United States of America; 4 Health Sciences Research Institute and School of Natural Sciences, University of California Merced, Merced, California, United States of America; University Freiburg, Germany

## Abstract

*Chlamydia pneumoniae* (*CP*) is an important human pathogen that causes atypical pneumonia and is associated with various chronic inflammatory disorders. Caspase-1 is a key component of the ‘inflammasome’, and is required to cleave pro-IL-1β to bioactive IL-1β. Here we demonstrate for the first time a critical requirement for IL-1β in response to *CP* infection. Caspase-1*^−/−^* mice exhibit delayed cytokine production, defective clearance of pulmonary bacteria and higher mortality in response to *CP* infection. Alveolar macrophages harbored increased bacterial numbers due to reduced iNOS levels in Caspase-1*^−/−^* mice. Pharmacological blockade of the IL-1 receptor in *CP* infected wild-type mice phenocopies Caspase-1-deficient mice, and administration of recombinant IL-1β rescues *CP* infected Caspase-1*^−/−^* mice from mortality, indicating that IL-1β secretion is crucial for host immune defense against *CP* lung infection. *In vitro* investigation reveals that *CP*-induced IL-1β secretion by macrophages requires TLR2/MyD88 and NLRP3/ASC/Caspase-1 signaling. Entry into the cell by *CP* and new protein synthesis by *CP* are required for inflammasome activation. Neither ROS nor cathepsin was required for *CP* infection induced inflammasome activation. Interestingly, Caspase-1 activation during *CP* infection occurs with mitochondrial dysfunction indicating a possible mechanism involving the mitochondria for *CP*-induced inflammasome activation.

## Introduction

*Chlamydia pneumoniae* (*CP*) is a widely prevalent [1] intracellular Gram-negative pathogen that causes upper respiratory infections and contributes to the development of chronic inflammatory conditions such as asthma [2], atherosclerosis [3], arthritis [4], and chronic obstructive pulmonary lung disease (COPD) [5].

In a mouse model of *CP* lung infection, effective host defense requires signaling through TLR/MyD88 [6] and NOD/Rip2 [7]. Toll-like receptor (TLR) 2 and TLR4 both use MyD88 to recognize *CP* [8], although TLR2 plays the larger role in host responses to *CP* infection [9]. MyD88 is also required for IL-1β signaling and *CP* infection has been shown to elicit strong IL-1β secretion in a number of experimental models [10], [11], [12]. In addition, alveolar macrophages and peripheral blood mononuclear cells obtained from COPD patients after *CP* infection secrete significantly higher amounts of IL-1β and lower amounts of IL-1R-antagonist, suggesting that IL-1β potentially mediates the pathogenesis of *CP* infection in COPD [13].

Secretion of IL-1β, a potent pyrogen that elicits a strong pro-inflammatory response [14], is tightly controlled by a diverse class of cytosolic complexes known as inflammasomes [15]. The NOD-like Receptor (NLR) family member NLRP3 forms cytosolic oligomers with apoptosis-associated speck like protein (ASC) in dendritic cells [16] and macrophages [17], triggering autocatalytic activation of caspase-1 [18]. Caspase-1, in turn, cleaves pro-IL-1β, producing mature IL-1β. Under normal circumstances, NLRP3 undergoes bipartite activation [15]. The first signal, often NF-kB activation, induces pro-IL-1β and NLRP3 expression. The second signal, any one of a variety of unrelated entities—particulate matter [19], crystals [20], aggregated β-amyloid [21], extracellular ATP [22], [23] and microbial toxins [24]—activates NLRP3. Exactly how these diverse cytosolic danger signals trigger the same inflammasome still remains unresolved and is the subject to intense research currently.

Here we show that caspase-1 dependent IL-1β secretion is critical for host defense in a mouse model of *C. pneumoniae* lung infection. Delayed cytokine production and reduced iNOS levels results in delayed bacterial clearance and increased mortality in caspase1*^−/−^* mice. Furthermore, administration of recombinant IL-1β to caspase-1*^−/−^* mice rescues the phenotype, while administration of the IL-1RA to wild type mice phenocopies caspase-1*^−/−^* mice. *CP* infection induced IL-1β production was dependent on TLR2/MyD88 signaling and required activation of the NLRP3/ASC/Caspase-1 inflammasome.

## Results

### Caspase-1 and IL-1β are critical for host innate immune defense against pulmonary CP infection

**Figure 1:**
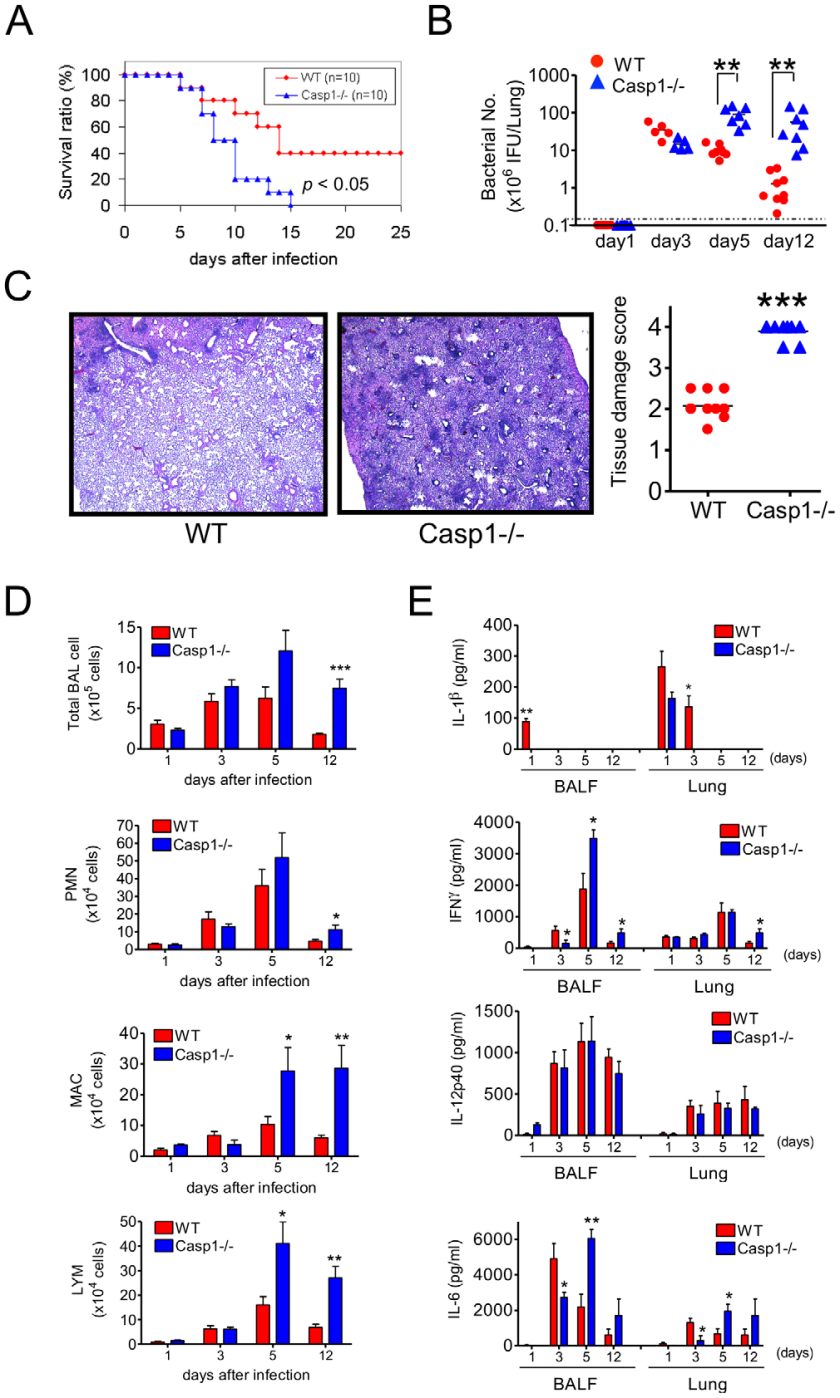
Casp1 plays a critical protective role during *CP* lung infection. (A) *Casp1^−/−^* mice or WT were infected intratracheally with 1.5×10^6^ inclusion forming units (IFU) of *CP* (n = 10). The Kaplan-Meier survival curve is shown. Statistical significance was determined by Fisher’s exact test. (B) Bacterial burden in infected (1×10^6^ IFU/mouse) WT and *Casp1^−/−^* lung homogenates was also quantified. Data shown are representative of three independent experiments. (C) Lungs were harvested 12 days after infection (1×10^6^ IFU/mouse), fixed in 10% buffered formalin, embedded in paraffin, sectioned, stained with hematoxylin and eosin (H&E, representative images shown) and scored for tissue damage. (D) BALF was harvested at days 1, 3, 5 and 12 following *CP* infection (1×10^6^ IFU/mouse) and the number of macrophages (MAC), polymorphonuclear cells (PMN) and lymphocytes (LYM), as well as the total cell number, was determined (n = 5∼9). (E) Cytokine (IL-1β, IFN-γ, IL-6, and IL-12p40) levels in both BALF and lung homogenates were determined using ELISA. Data for all experiments shown represent at least two independent experiments. Note on statistical significance: * p<0.05, ** p<0.01, *** p<0.001 (Student’s t test used unless otherwise noted).

To determine the role of Caspase-1 (Casp1) in host defense against *CP*, we infected *Casp1^−/−^* mice intratracheally with 1.5×10^6^ inclusion forming units (IFU) of CP. At this infectious dose, *Casp1^−/−^* mice exhibit significantly greater mortality (Figure 1A), pulmonary bacterial titers (Figure 1B) and inflammatory lung damage (12 days post-infection) compared to wild-type C57BL/6 (WT) mice (Figure 1C). Examination of the bronchoalveolar lavage fluid (BALF) and lung homogenates from *CP* infected *Casp1^−/−^* mice revealed significantly greater leukocyte recruitment, particularly of macrophages and lymphocytes, at days 12 post infection compared to WT mice (Figure 1D). Given that innate immune defenses limit *CP* replication and colonization and that Casp1 is required for processing of pro-IL-1β [25], we hypothesized that cytokine production would differ in *Casp1^−/−^* and WT mice. IL-1β was undetectable in the BALF (at all time points) and in homogenates (days 5 and 12) in *Casp1^−/−^* mice (Figure 1E). IL-1β was detected only at the earliest time in the BALF of WT mice. Of note, IL-1β was detected by ELISA on day 1 in *Casp1^−/−^* lungs. This is most likely pro-IL-1β released as a result of cell lysis during tissue homogenization.

Early on at days 1 and 3 post-infection, we observed a significant delay in IFN-γ and IL-6 production in *CP* infected *Casp1^−/−^* mice compared to WT animals, but these cytokines were significantly elevated on days 5 and continued trending higher on day 12 (Figure 1E). Notably, *CP* infected *Casp1^−/−^* and WT mice exhibited no significant differences in IL-12p40 secretion at any time point examined (Figure 1E). Taken together, these results indicate that Casp1 plays a key role in initiation of early inflammatory responses that lead to bacterial clearance in the lungs and survival from infection.

**Figure 2:**
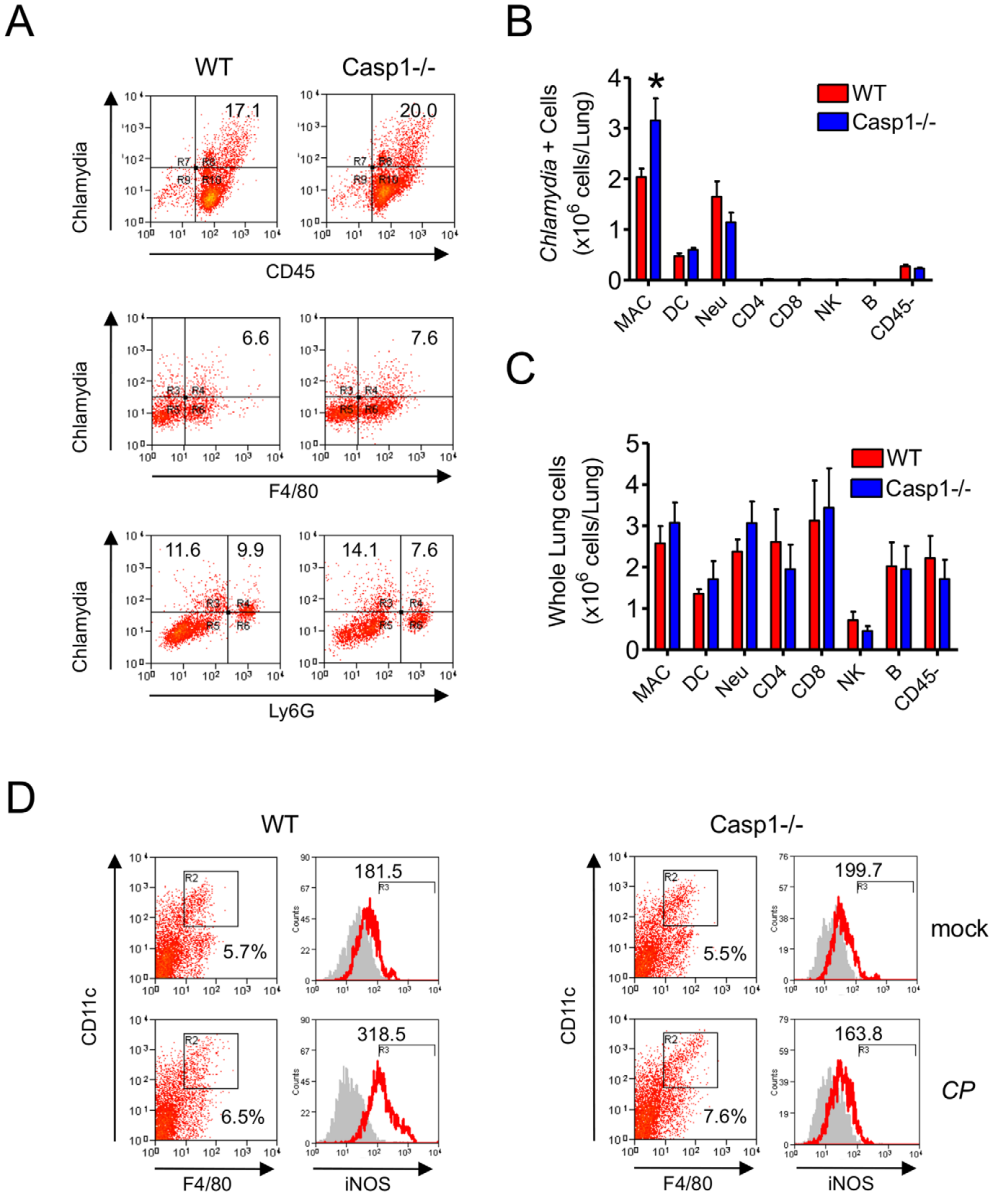
*Casp1^−/−^* alveolar macrophages do not produce iNOS in response to *CP* infection. (A and B) Compared to WT, *Casp1^−/−^* phagocytes contain more *Chlamydia* and macrophages are the principal *CP* harboring cell type. Single-cell suspensions from *CP* infected lungs of WT and *Casp1^−/−^* were prepared 12 days following infection. Cells were then stained for characteristic leukocyte markers and stained for intracellular *CP* with a FITC conjugated anti-*Chlamydia* monoclonal antibody (mAb), and analyzed by flow cytometry to determine which cell types contain *CP*. Representative flow cytometry data plots of CD45+ cells, F4/80+ cells (CD11c+ gated), and Ly6G+ (CD11b+ gated) cells are shown. (C) Also shown are the proportions of total lung leukocytes that contain *CP* in WT and *Casp1^−/−^* mice and the absolute numbers of leukocytes in *CP* infected lungs from WT and *Casp1^−/−^* mice. (D) iNOS expression in alveolar macrophages (CD11c+, F4/80+) 2 days post-infection. Representative histograms are shown and the mean fluorescence intensity (MFI) is indicated. Data for all experiments shown represent at least two independent experiments. Note on statistical significance: * p<0.05 (Student’s t test used unless otherwise noted).

We next sought to determine the predominant lung cell type infected by *CP* in *Casp1^−/−^* mice. Since *CP* is an obligate intracellular pathogen, we analyzed lung cells by intracellular flow cytometry after infection by *CP* in *Casp1^−/−^* mice *vs*. WT mice. *CP* was predominantly found in alveolar macrophages (AM) and, to a lesser degree, in neutrophils and dendritic cells (DC) on day 12 (Figure 2A–B). Specificity of staining for *CP* is shown using isotype control (Figure S1). There were relatively more AM in infected *Casp1^−/−^* mice than their wild-type counterparts (Figure 2C), suggesting that AM could be a reservoir of bacterial replication after pulmonary infection in *Casp1^−/−^* mice.

We next wished to determine if AM isolated from *CP* infected *Casp1^−/−^* mice exhibited an immune defect compared to cells from infected WT mice. Indeed, we found that, unlike WT AM, *Casp1^−/−^* AM did not induce iNOS following *CP* infection at day 2 (Figure 2D). Nitric oxide (NO) produced by macrophages after cell activation by IFN-γ hampers the growth of *CP* [26], and these findings are consistent with the delayed IFN-γ production observed in *Casp1^−/−^* mice (Figure 1C), supporting a model whereby IL-1β secretion by AM induces early IFN-γ that, in turn, activates AM at the infection site to induce iNOS and clear *CP*.

**Figure 3:**
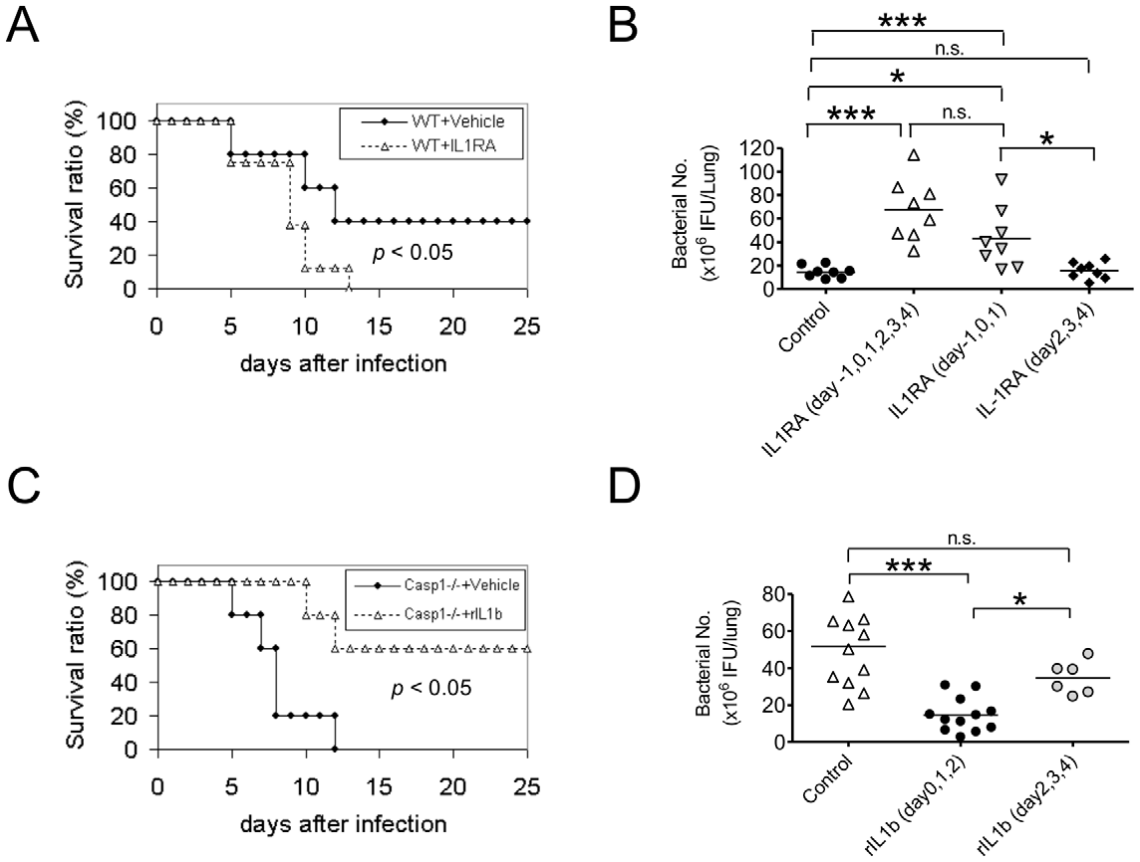
IL-1 signaling is crucial for host survival and bacterial clearance during *CP* lung infection. (A and B) WT mice were daily given an IL-1RA (500 µg, i.p.) or vehicle control and then infected with *CP* (1.5×10^6^ IFU/mouse). The Kaplan-Meier survival curve is shown. Bacterial burden in lung homogenates was determined for IL-1RA and vehicle control treated mice 5 days post infection with 1×10^6^ IFU (n = 8). (C and D) *Casp1^−/−^* mice were treated with rIL-1β (8 ng, i.p.) daily for 3 days or vehicle control and then infected with *CP* (1.5×10^6^ IFU/mouse) (n = 5). The Kaplan-Meier survival curve is shown. Bacterial burden in lung homogenates of rIL-1β and vehicle control treated mice 5 days after infection with 1×10^6^ IFU (n = 6–12). Data for all experiments shown represent at least two independent experiments. Note on statistical significance: one-way ANOVA with Tukey’s post-hoc test - * p<0.05, ** p<0.01, *** p<0.001.

To verify that Casp1 activation exerted its protective role via activating IL-1β secretion (as opposed to IL-18), wild-type mice were injected daily with either IL-1 receptor antagonist (IL-1RA) or a control vehicle. Like *Casp1^−/−^* mice, treatment of WT mice with IL-1RA on days -1, 0 and 1 relative to *CP* infection caused a significant increase in mortality (Figure 3A) and in bacterial load (Figure 3B) in the lung (at day 5, *p*<0.001) compared to vehicle control. Treatment timing was crucial, as early treatment (days −1 to 1 or days −1 to 4) with IL-1RA, but not later dosing with IL-1RA (days 2 to 4), resulted in impaired bacterial clearance at day 5 (Figure 3B), suggesting that the immunoprotective effects of IL-1β occur early during lung infection with *CP*.

To further verify the role of IL-1β signaling in host defense against *CP* infection, we performed a complementary experiment where *CP* infected *Casp1^−/−^* mice were injected with either recombinant IL-1β (rIL-1β) or a control vehicle. Early rIL-1β treatment during infection (i.e. days 0, 1, and 2 post-infection) rescued *Casp1^−/−^* mice, restored survival (Figure 3C) and reduced lung bacterial load (Figure 3D). Yet, mice treated later (days 2, 3 and 4 post-infection) showed significantly increased bacterial counts (*p*<0.05) relative to early-treated mice (Figure 3D), indicating that IL-1β secretion is critical for initial host immune responses that limit bacterial proliferation in the lung.

### Macrophage TLR2/MyD88 and NLRP3/ASC/Caspase-1 are required for IL-1β secretion in response to CP

**Figure 4:**
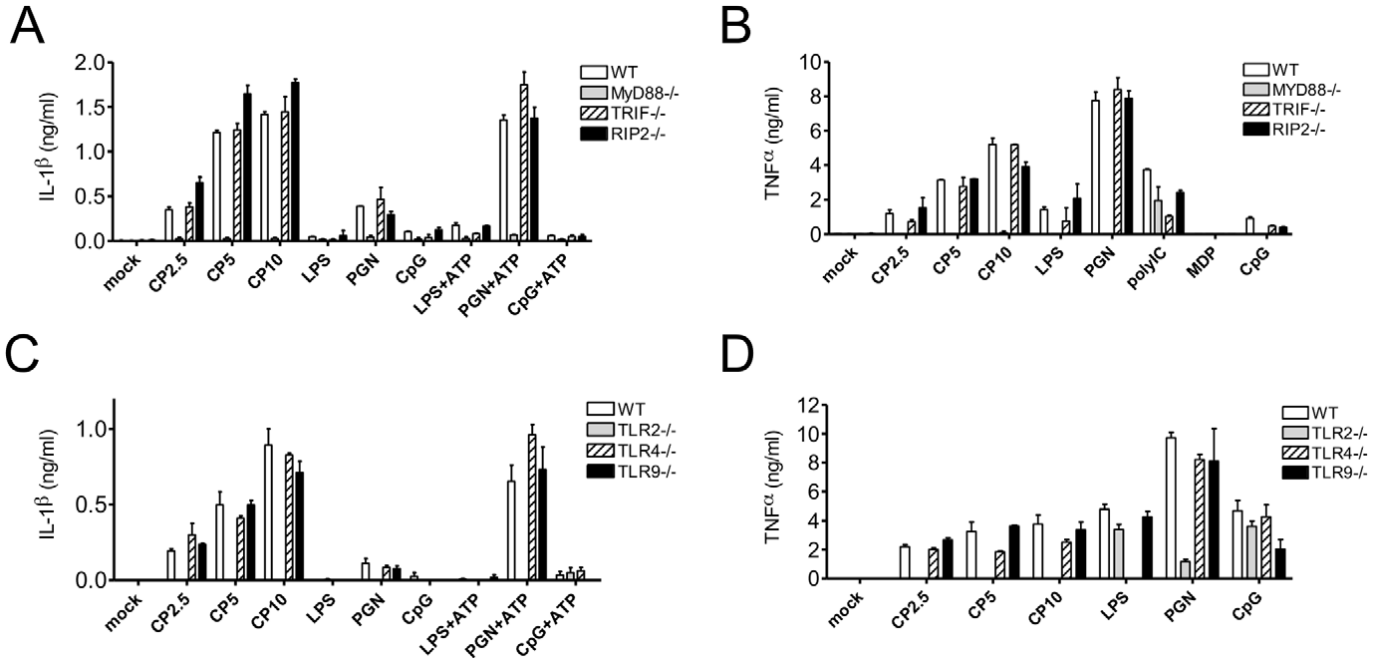
TLR2/MyD88 is indispensable for *CP*–induced IL-1β and TNFα production by macrophages. (A–B) WT, *MyD88^−/−^*, *Trif^−/−^*, and *Rip2^−/−^* BMDM were treated with live *C. pneumoniae* (MOI 2.5, 5, 10), LPS (1 µg/ml), PGN, poly I:C, muramyl dipeptide (MDP), and CpG DNA and as indicated, a proportion of the cells were also treated with 5 mM ATP for the final 2 h of culture. The culture supernatants were assessed for IL-1β and TNFα production by ELISA. (C–D) WT, *Tlr2^−/−^*, *Tlr4^−/−^*, and *Tlr9^−/−^* BMDM were treated with live *C. pneumoniae* (MOI 2.5, 5, 10), LPS (1 µg/ml), PGN, and CpG DNA and as indicated, a proportion of the cells were also treated with 5 mM ATP for the final 2 h of culture. The culture supernatants were assessed for IL-1β and TNFα production by ELISA. Data for all experiments shown represent at least three independent experiments.

**Figure 5:**
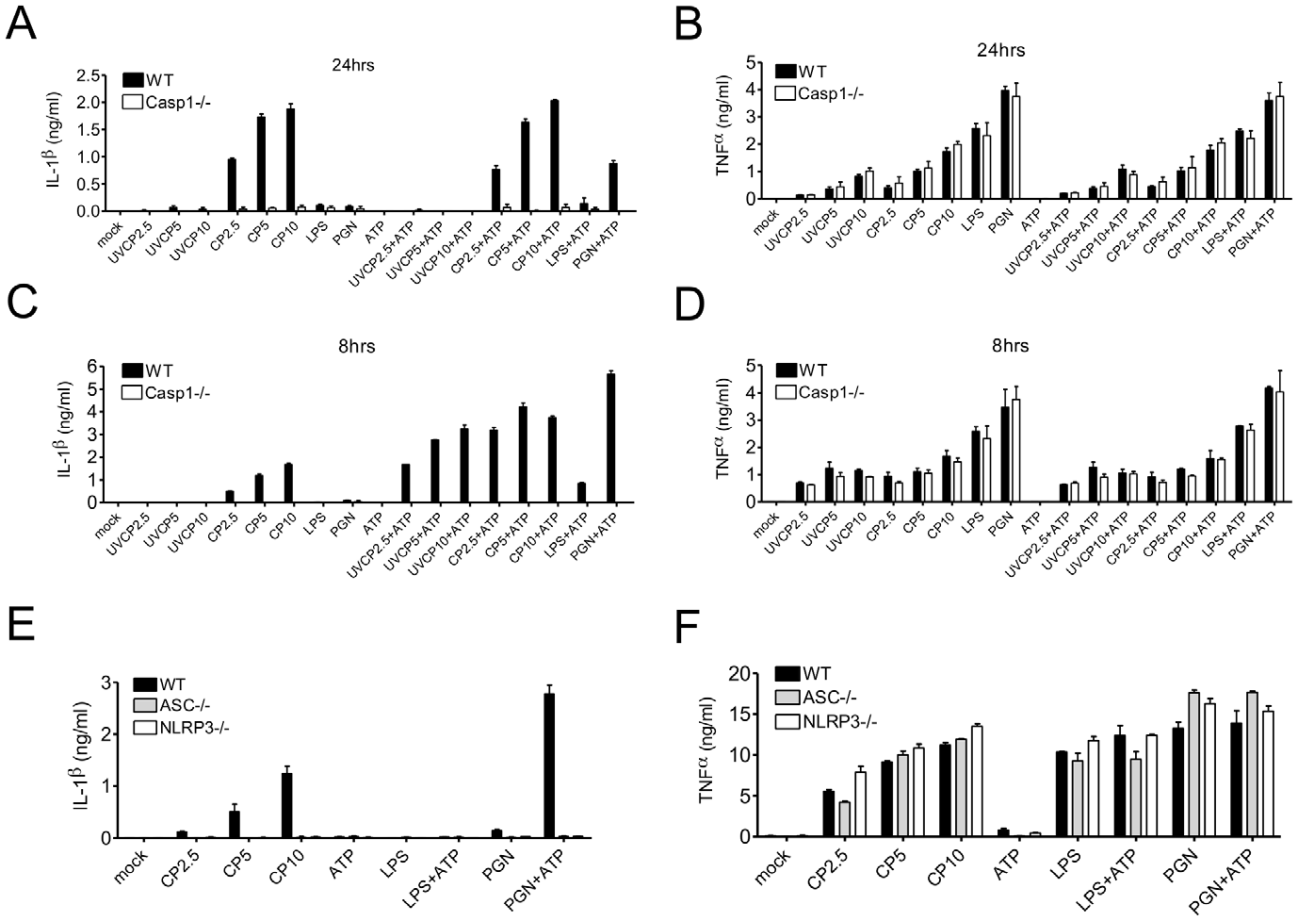
Macrophage NLRP3/ASC/Caspase-1 inflammasome are required for IL-1β secretion in response to *CP*. (A, B, C, and D) IL-1β and TNFα concentrations were measured in the culture supernatants of WT and *Casp1^−/−^* BMDM treated for 24 h or 8 h with *CP* (MOI 2.5, 5, 10), UVCP (MOI 2.5, 5, 10), lipopolysaccharide (LPS, 1 µg/ml) or peptidoglycan (PGN, 10 µg/ml). As indicated, a proportion of cells were also treated with 5 mM ATP for the final 2 h of culture. (E and F) IL-1β and TNFα concentrations in the culture supernatants of WT, *Asc^−/−^*, and *Nlrp3^−/−^* BMDM were measured 24 hours after treatment with UVCP (MOI 2.5, 5, 10), live *CP* (MOI 2.5, 5, 10), LPS or PGN. As indicated, a proportion of cells were also treated with 5 mM ATP for the final 2 h of culture. Data shown are representative of at least three independent experiments. Note on statistical significance: * p<0.05, ** p<0.01, *** p<0.001 (Student’s t test used unless otherwise noted).

As AM are one of the major *CP*-harboring cells in lung, we sought to elucidate the mechanism of *CP* induced IL-1β production and secretion by macrophages *in vitro*. Western analysis clearly indicated that *CP* infection of macrophages induced activation of caspase-1 and secretion of cleaved IL-1β (Figure S2). To determine which signaling pathway played a role in *CP* induced pro-IL-1β production, we infected MyD88-, TRIF- or RIP2-deficient BMDM with *CP* for 24 hours and then measured IL-1β secretion. MyD88, but neither TRIF nor RIP2 signaling, was required for IL-1β secretion in response to *CP* (Figure 4A–B). Further investigation revealed that pathogen sensing by TLR2/MyD88 is of central importance to induction of both IL-1β and TNFα secretion by BMDM during *CP* infection (Figure 4C–D). At 8 hours and, more so, at 24 hours post-infection, cultured BMDM infected with *CP* secreted IL-1β in a Casp1-dependent manner (Figure 5A and 5C). On the contrary, neither 8 hr nor 24 hr treatment of BMDM with UV-killed *CP* (UVCP) stimulated IL-1β secretion (Figure 5A and 5C), indicating that active *CP* infection is required to induce IL-1β secretion. *Casp1^−/−^* BMDM demonstrated wild-type phagocytic capability (Figure S3A) and TNFα production (Figure 5B and 5D). Additionally, bacterial replication in *Casp1^−/−^* BMDM was normal (Figure S3B). Though UVCP was unable to induce IL-1β secretion by BMDM, we hypothesized that UVCP induced pro-IL-1β production. To test this, we treated BMDM with UVCP and then exposed them to a high extracellular concentration of ATP (5 mM), a stimulus known to activate Casp1 and IL-1β release via the NLRP3 inflammasome. Interestingly, BMDM treated with UVCP for 6 hours and then exposed to ATP for 2 additional hours induced IL-1β secretion (Figure 5C). ATP treatment also increased IL-1β secretion in BMDM after a 6 hours (but not 24 hours) infection with live *CP* (Figure 5A and 5C); but BMDM treated with UVCP for 24 hours and then challenged with ATP did not secrete IL-1β (Figure 5A). Consistently, UVCP- and LPS-induced pro-IL-1β was decreased at 18 h and 24 h (Figure S4), suggesting that pro-IL-1β is degraded if Casp1 is not activated within a narrow time window.

Since the NLRP3 inflammasome activates Casp1 in response to a wide array of stimuli, we hypothesized that *CP* may also induces IL-1β secretion via NLRP3. *Nlrp3^−/−^* and *Asc^−/−^* BMDM infected with live *CP* secreted dramatically less IL-1β (Figure 5E). Nevertheless, *Nlrp3^−/−^* and *Asc^−/−^* BMDM retained their ability to secrete TNFα at WT levels (Figure 5F). This indicates that *CP* infection of macrophages induces IL-1β secretion via the NLRP3/ASC inflammasome.

### NLRP3 activation by CP requires cellular entry and new protein synthesis and is independent of ROS production

**Figure 6:**
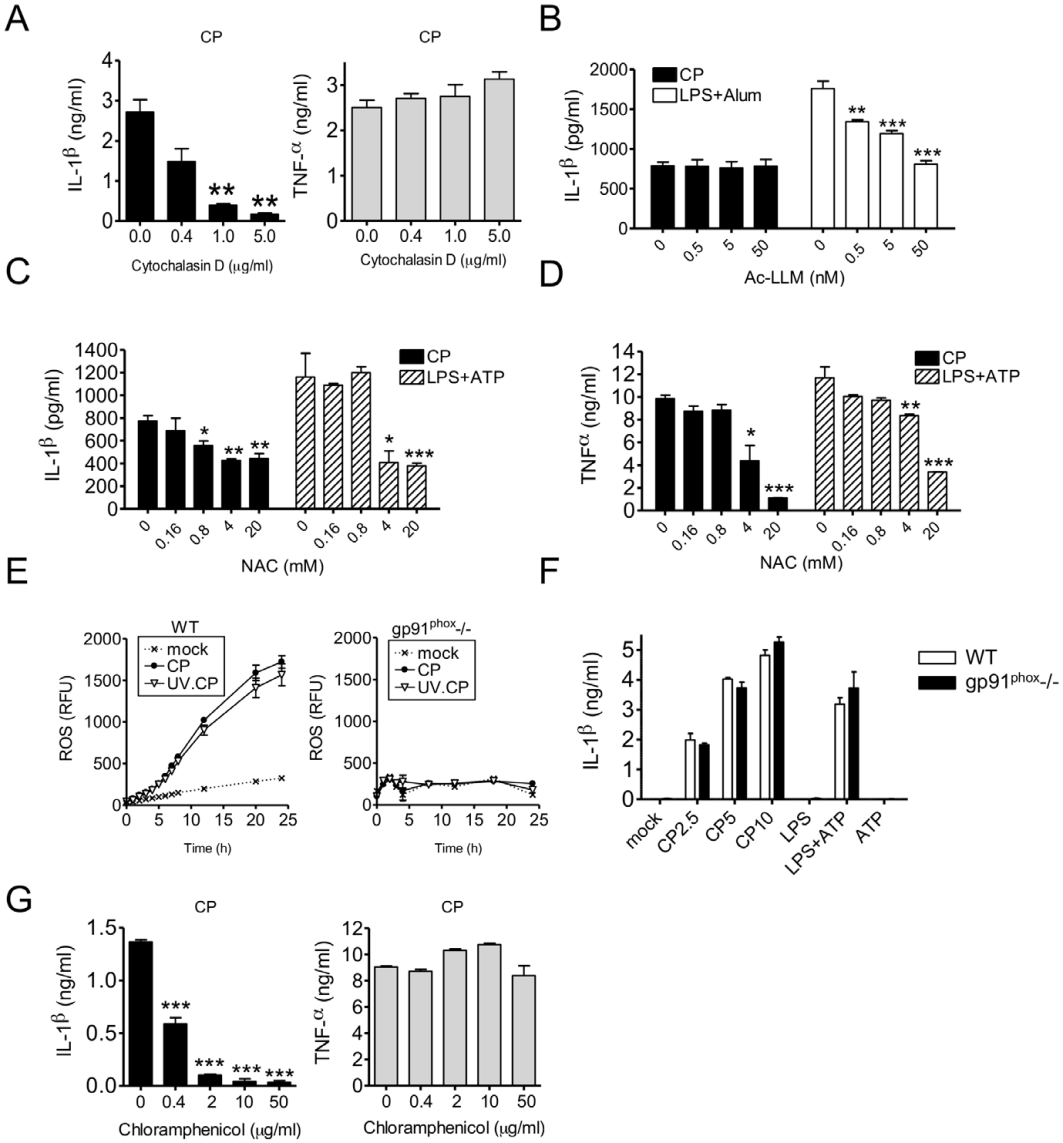
Phagocytosis and bacterial de novo protein synthesis are necessary to activate the NLRP3 inflammasome in *CP* infected macrophages. (A) IL-1β (black bars) and TNFα (gray bars) secretion by *CP* infected (MOI 10, 24 h) BMDM in the presence or absence of cytochalasin D was quantified using ELISA. (B) *CP* activation of the NLRP3 inflammasome in macrophages is cathepsin independent. Using ELISA, IL-1β concentration in culture supernatants of *CP* infected (MOI 10, 24 h) BMDM was determined in the presence of increasing amounts of Ac-LLM. Also, LPS-primed (1 µg/ml, 8 h) BMDM treated with Alum (130 µg/ml, final 2 h of culture) were given increasing amounts of Ac-LLM. (C and D) Antioxidant (N-acetylcysteine, NAC) treatment does not specifically inhibit IL-1β secretion. ELISA was used to determine IL-1β and TNFα concentration in the culture supernatants of *CP* (MOI 10, 24 h) infected BMDM and LPS-primed (8 h), ATP treated (5 mM, final 2 h culture) BMDM in the presence of increasing doses of NAC. (E) A fluorometric assay was used to quantitate ROS production by BMDM in response to UVCP or live *CP*. (F) gp91^phox^ defected BMDM were treated with live *CP* (MOI 2.5, 5, 10, 24 h), or alternatively, 6 h after LPS priming BMDM were then treated with ATP (5 mM) and cultured for an additional 2 h. Culture supernatant was then collected and IL-1β concentration was measured by ELISA. (G) ELISA was used to determine IL-1β and TNFα concentration in the culture supernatants of *CP* (MOI 10, 24 h) infected BMDM in the presence of increasing doses of chloramphenicol. Data shown are representative of two or more independent experiments. Note on statistical significance: * p<0.05, ** p<0.01, *** p<0.001 (Student’s t test used unless otherwise noted).

To further understand the mechanism by which live *CP* activates the NLRP3 inflammasome in macrophages, we tested whether *CP* cell entry is required for IL-1β secretion. In the presence of cytochalasin D, an inhibitor of actin polymerization and phagocytosis, *CP*-induced IL-1β secretion (but not TNFα secretion) by BMDM was significantly reduced in a dose dependent manner, suggesting that NLRP3 activation requires bacterial uptake by the host cell (Figure 6A). Cytochalasin D did not affect LPS + ATP induced IL-1β production (data not shown).

We next examined whether phagosomal cathepsin B activity plays a role in IL-1β secretion in response to *CP* infection. An inhibitor of cathepsins B and L, N-Acetyl-Leu-Leu-Met-al (Ac-LLM), did not alter IL-1β secretion induced by *CP*, but did reduce alum induced IL-1β secretion from LPS-primed BMDM (Figure 6B). Therefore, Cathepsin B activity likely does not play a significant role in *CP*-induced inflammasome activation.

To test whether ROS play a role in inflammasome activation during *CP* infection, we treated *CP* infected BMDM with the antioxidant *N*-acetyl-L-cysteine (NAC). Although we observed that NAC attenuates *CP* induced IL-1β secretion, we also found that this antioxidant reduces TNFα production (Figure 6C). A concomitant reduction of both IL-1β and TNFα secretion was also observed when LPS-primed BMDM were pre-treated with NAC before ATP addition (Figure 6D). Indeed, others have also observed NF-κB inhibition by antioxidants [27]; therefore, we believe this non-specific effect precludes using NAC to reach any conclusions about ROS in *CP-* induced NLRP3 activation.

In an effort to clarify if ROS plays any role in *CP-* induced IL-1β production, we measured directly the amount of ROS produced during live *CP* infection and during treatment with UVCP. Remarkably, UVCP displayed a ROS generation profile nearly identical to that of live *CP* in macrophages (Figure 6E) and that the ROS generation was completely dependent on the NADPH phagocyte oxidase pathway, as macrophages defective in this pathway (*gp91^phox−/−^*) did not make ROS in response to *CP* infection (Figure 6E) [28]. Finally, we assessed the ability of *CP* infection to induce IL-1β secretion in *gp91^phox−/−^* BMDM. Not surprisingly, given the previous data, *gp91^phox−/−^* BMDM were unaltered in their ability to secrete lL-1β in response to *CP* infection (Figure 6F). Additionally, LPS plus ATP induced IL-1β secretion was also not affected. Since live *CP* but not UVCP treatment induces IL-1β secretion but both induce same amount of ROS, and the lack of phagocytic ROS in *gp91^phox−/−^* BMM did not alter IL-1β secretion, we conclude that ROS generated during *CP* infection are not required for NLRP3 inflammasome activation.

To shed more light on exactly how *CP* activates NLRP3, we next set out to determine the role of bacterial protein synthesis in *CP-*induced IL-1β secretion. Treatment of *CP* infected BMDM with the antimicrobial chloramphenicol, an inhibitor of the bacterial ribosome, led to nearly complete inhibition of IL-1β secretion, without affecting TNFα production (Figure 6G). As expected, *CP* inclusion formation and size were dose dependently inhibited by chloramphenicol treatment (Figure S5A–B). This result indicates that *de novo* protein synthesis by *CP* is necessary for Casp1 activation in infected BMDM. The addition of chloramphenicol at a similar concentration did not affect LPS + ATP induced IL-1β secretion (data not shown).

### CP infection in macrophages induces mitochondrial dysfunction

**Figure 7:**
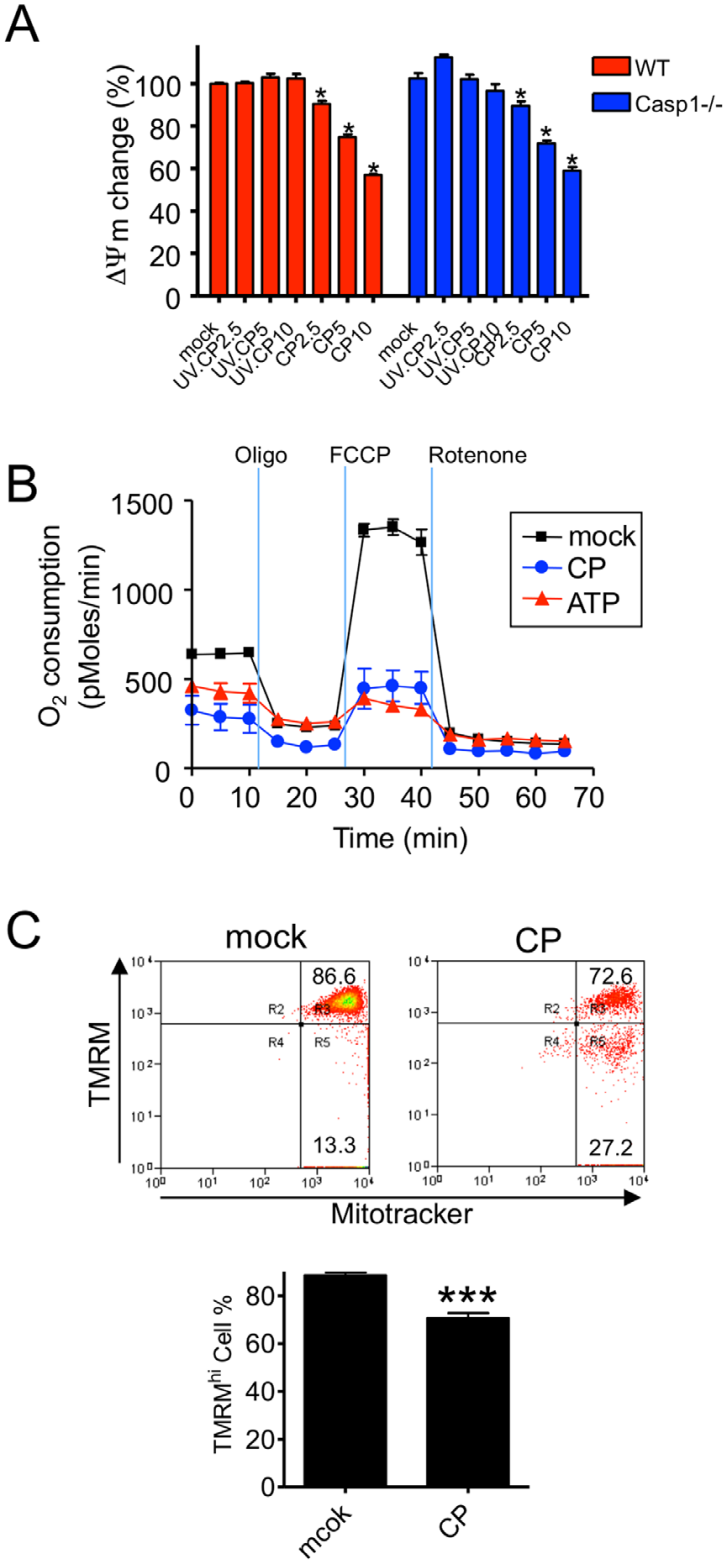
*CP* infection induces mitochondrial dysfunction. (A) WT and *Casp1^−/−^* BMDM were treated with UVCP (MOI 2.5, 5, 10), or live *C. pneumoniae* (MOI 2.5, 5, 10) and then examined for TMRM incorporation. (B) Oxygen consumption rate (OCR) was measured in macrophages (MCL) infected with *CP* (MOI 10, 16 h) or treated with ATP (5 mM, 1 h) by using an XF24 Extracellular Flux Analyzer. After incubation in basal media, 1 µM oligomycin, 1 µM carbonyl cyanide-p-trifluoromethoxyphenylhydrazone (FCCP), and 1 µM rotenone were each sequentially injected as indicated, and the response was monitored. (C) BALF was collected 24 h post *CP* infection in WT mice. Cells were stained with anti-F4/80 mAb and labeled with TMRM and Mito tracker green for 30 min. Mitochondrial membrane potential in alveolar macrophage (F4/80+, SSC-hi) were analyzed by flow cytometry. Statistical significance was determined by Student’s *t* test in comparison to mock control (n = 4). Data shown are representative of three or more independent experiments. Statistical significance was determined by Student’s *t* test in comparison to non-treated cells (* p<0.05, ** p<0.01, *** p<0.001).

Two recent reports indicated that the mitochondrial dysfunction might be an important link in NLRP3 activation, while the exact mechanism how the mitochondria plays a role in this activation must await additional mechanistic studies [29], [30]. Since both ROS and cathepsin did not appear to play a role in *CP* infection induced inflammasome activation, we decided to look at the role of mitochondria. We determined the effect of *CP* infection on inner mitochondrial membrane potential (ΔΨm), a readout of mitochondrial function. Infection with live *CP*, but not UVCP treatment, resulted in a significant decrease in ΔΨm (Figure 7A) as measured by a tetramethyl rhodamine methyl ester (TMRM) incorporation assay in BMDM, implicating a potential role of the mitochondria in NLRP3 inflammasome activation and IL-1β release. Utilizing *Casp1^−/−^* BMDM, we found that *CP* induced mitochondrial depolarization independent of Casp1 (Figure 7A), suggesting that this observation is independent of pyroptosis, which by definition is Casp1-dependent. In an effort to further characterize the mitochondrial dysfunction induced by *CP* infection, we analyzed the rate of O_2_ consumption in macrophages. Impressively, either *CP* infection or the addition of ATP drastically reduced the rate of oxygen consumption in these cells, indicating a severe mitochondrial dysfunction under these conditions (Figure 7B). Furthermore, we determined whether mitochondrial depolarization was observed *in vivo* following *CP* infection. *CP* infection significantly decreased TMRM incorporation in AM but not non-metabolic labeling of mitochondria (Mito tracker) (Figure 7C). In addition to *in vitro* data, mitochondrial dysfunction was observed *in vivo* following *CP* infection. Thus it seems probable that *CP* infection induced inflammasome activation involves mitochondrial dysfunction and that this dysfunction is independent of caspase-1 activity.

## Discussion

We show here that Casp1-dependent IL-1β secretion is critically required for host defense against *CP* lung infection. *Casp1^−/−^* mice displayed delayed pulmonary bacterial clearance leading to increased mortality compared to WT mice. Macrophages play a key role in this process, as they respond to *CP* via the NLRP3 inflammasome. *Casp1^−/−^* mice showed delayed IFN-γ production and defective iNOS activation in *Casp1^−/−^* AM, consistent with reports demonstrating a critical role of IFN-γ and iNOS in clearing *CP* infection [26].

In our model of *CP* lung infection, IL-1β plays a critical role in orchestrating a successful host defense against infection. In addition to *Casp-1^−/−^* mice, blockade of IL-1β signaling using the IL-1RA resulted in increased mortality to a *CP* infection. Indeed, early IL-1β signaling proved to be critical as rIL-1β given to caspase-1*^−/−^* was able to rescue these mice from a lethal *CP* infection, but only when given at the earliest time points. These data also highlight that it is unlikely that IL-18 plays a significant role during *CP* infection.

IL-1β has been known to be an important initiator of acute phase inflammatory responses to infections [31] and more recently, found to play an critical role in establishing a Th17 response [32]. In our model, we found that in caspase-1*^−/−^* mice IFN-γ production was significantly delayed, resulting in poor bacterial clearance. It has been well established that IFN-γ is required for proper clearance of *CP* in mice [33], [34]. We also found that iNOS was not induced in alveolar macrophages in caspase-1*^−/−^* mice at day 2. While alveolar macrophages are a major site of *CP* replication, they also play a critical role in bacterial clearance. Importantly, iNOS is critically important for *CP* clearance and can be induced by both IFN-γ and IL-1β [26], [35]. Therefore, the defective iNOS induction in alveolar macrophages early during infection (day 2) most likely plays a significant role in the defective bacterial clearance.

IL-1β was found to be critically important for several other bacterial infections, including *S. aureus*, *B. anthracis*, and *M. tuberculosis*. Miller et al. found that mice lacking IL-1β developed large skin lesions due to a reduction in neutrophil recruitment during a cutaneous *S. aureus* infection [36]. In another study, Moayeri et al. found similar results indicating the requirement of IL-1β for proper neutrophil recruitment against *B. anthracis* infection [37]. Finally, IL-1β*^−/−^* mice showed greatly increased mortality to *M tuberculosis* infection [38]. Interestingly, these mice did not have any defects in nitrite production or IFN-γ or cellular recruitment, thus the mechanism by which IL-1β acts is unknown in this model. Taken together and including our data, it is clear that IL-1β can play a critical role in the host defense against a bacterial infection.

In our study we found that *CP* infection induced IL-1β processing through TLR2/MyD88 signaling and activation of the NLRP3 inflammasome. This process required live bacteria, as UVCP did not induce IL-1β secretion without additional stimuli such as ATP. Additionally, entry into the cells was required for inflammasome activation as was active protein synthesis in the bacteria. *CP* does possess a type III secretion system and it is possible that it might be involved in NALP3 inflammasome activation. However, as there is no genetic manipulation available yet for *CP* and the type III secretion inhibitors proposed to be specific against *CP* [39] have many off target and non-specific inhibitory effects, the direct role of type III secretion in NALP3 activation can not be assessed currently.

Similar to our findings, He *et al.* recently reported that *CP* required TLR2 and the NLRP3/ASC inflammasome for IL-1β production [40]. However, unlike our study, they were unable to determine a role for IL-1β in the model they used. These investigators used IL-1R deficient mice, and these mice showed little difference if any on the course of infection. We demonstrate for the first time the critical role of IL-1β in host defenses against *CP* lung infection. While we used caspase-1*^−/−^* mice, which would affect both IL-1β and IL-18 production, our reconstitution experiment with IL-1β in caspase-1*^−/−^* mice, plus the use of the IL-1RA in WT mice, clearly showed that IL-18 is dispensable in the host response to *CP* infection. A source of the differences between our results and those by He *et al.* could be due to different models used, including different *CP* strains used in these two studies (A03 strain by He *et al.* as opposed to CM-1 strain used in our study), and a much higher dose of *CP* (2×10^7^) used by He *et al*. versus (1×10^6^) used in our study. *CP* strain-specific differences likely led to the much milder lung infection seen in the study by He *et al*. and perhaps this accounts for the large differences found between our study and theirs regarding infection course and mortality following murine *CP* infection.

It is currently not clear how *CP* infection activates the NLRP3 inflammasome. A wide range of cytosolic danger signals have been shown to lead to activation of the NLRP3 inflammasome. It is believed that three broad physiological changes—reactive oxygen species (ROS) generation, potassium cation (K+) efflux, or lysosomal leakage—activate the NLRP3 inflammasome [41], while direct mechanistic studies as to how they activate NLRP3 are yet to be provided. Furthermore, these three proposed models of NLRP3 activation are not even reconciled with one another and no model that offers a unifying paradigm exists. Our data indicates that ROS is not involved in activating the NLRP3 inflammasome during *CP* infection. Even though UVCP and *CP* induced similar amounts of ROS in macrophages, UVCP does not elicit IL-1β secretion while live *CP* does. Moreover, our results call in to question results from studies that use the antioxidant N-acetyl cysteine (NAC). Though this agent was found to reduce IL-1β secretion, it also caused a concomitant reduction in TNF-α production, indicating that NAC likely affects pro-IL-1β production via NF-κB. Lastly, in agreement with previous studies [42], we report here that macrophages deficient in NADPH oxidase activity, and thus in phagocytic ROS production (*gp91^phox−/−^*), exhibit normal IL-1β production in response to NLRP3 stimuli, refuting the role of ROS in NLRP3 activation. However these data only determined the role for cellular derived ROS, not mitochondrial derived ROS. Recent publications have found an important role for mitochondrial ROS in NLRP3 activation, indicating an important role for this organelle [29], [30].

The lysosome rupture model also does not seem to be mechanism of *CP* infection induced NLRP3 inflammasome activation, as the cathepsin inhibitor that we used had no effect on *CP*-induced IL-1β production. This observation is different than those reported by He *et al.* who observed that cathepsin activity and lysosomal acidification both play a role in *CP-*induced IL-1β secretion. However, the inhibitors used in that study, CA-074Me (a cathepsin B and L inhibitor) and bafilomycin A (a lysosomal acidification inhibitor), both have off-target effects (as do most pharmacological inhibitors) [43], [44]. Additionally, *CP* is a small infectious elementary body (EB); 300–600 nm diameter compared to other intracellular bacteria, and so internalization of the EB is unlikely to exceed the capacity of the phagolysosome. As part of the *CP* life cycle, infectious EB converts to the vegetative reticulate body (RB), which forms inclusion bodies in the host cell phagosome (6.0–7.4 µm diameter). Though these inclusions might be large enough to cause vesicle rupture, *Chlamydia* are known to actively inhibit the process of phagolysosomal fusion [45]. So even if *CP*-containing phagosomes ruptured, lysosomal enzymes would ostensibly not be present. Also, we found that *CP* is able to induce IL-1β secretion in the presence of a specific cathepsin B inhibitor, Ac-LLM, further arguing against lysosomal degradation as the means by which *CP* activates the NLRP3 inflammasome.

Two recent papers have identified mitochondrial dysfunction as being involved in the activation of the NLPR3 inflammasome, especially in relation to autophagy. Zhou et al. found that both ROS generation and inflammasome activation are suppressed when mitochondrial activity is dysregulated by inhibition of the voltage-dependent anion channel [30]. Nakahira et al. also found that mitochondrial dysfunction played a role in inflammasome activation, and that mitochondrial DNA might play a role in this [29]. To this end we investigated the effect of *CP* infection on mitochondrial function. Our results indicated that both *CP* infection and the commonly used inflammasome activator LPS plus ATP resulted in mitochondrial dysfunction as measured by a reduction in mitochondrial membrane potential and reduced O_2_ consumption. Identifying mitochondria as a player in NLRP3 inflammasome induction could help explain the many differing pathways that result in NLRP3 activation. Both ROS and cathepsins released from the lysosome can affect mitochondrial membrane potential, as can K^+^ levels in the cell [46], [47], [48] [49]. With the addition of a bacterial infection to this mix, the role that mitochondria might play in inflammasome activation remains an important subject and may hold the key to allow us to understand the mechanism of NLRP3 activation.

## Materials and Methods

### Ethics Statement

All experiments were performed according to the guidelines and approved protocols (IACUC #2097) of the Cedars-Sinai Medical Center Institutional Animal Care and Use Committee and were housed under specific pathogen free conditions. Cedars-Sinai Medical Center is fully accredited by the Association for Assessment and Accreditation of Laboratory Animal Care (AAALAC International) and abides by all applicable laws governing the use of laboratory animals. Laboratory animals are maintained in accordance with the applicable portions of the Animal Welfare Act and the guidelines prescribed in the DHHS publication, Guide for the Care and Use of Laboratory Animals.

### Mice

*Casp1^−/−^* mice [50] were kindly provided by Dr. Richard Flavell (Yale Univ, New Haven, CT). *Nlrp3^−/−^* mice and *Asc^−/−^* mice [23] were generously provided by Dr. Katherine Fitzgerald (University of Massachusetts Medical School, Worcester, MA). C57BL/6, *gp91^phox−/−^* and *Trif^−/−^* mice were obtained from Jackson Labs. *MyD88^−/−^*, *Rip2^−/−^*, *Tlr2^−/−^*, *Tlr4^−/−^*, and *Tlr9^−/−^* (Naiki et al., 2005; Shimada et al., 2009) mice were maintained according to Cedars-Sinai Medical Center Institutional Animal Care and Use Committee guidelines. All mice were used at 8-12 weeks of age. *Casp1^−/−^* and *MyD88^−/−^* mice were backcrossed for eight generations, *Tlr9^−/−^* and *Rip2^−/−^* mice were backcrossed for 10 generations, *Tlr2^−/−^* and *Tlr4^−/−^* mice were backcrossed for 16 generations, *Gp91^phox−/−^* mice were backcrossed for 13 generation, and *Nlrp3^−/−^* and *Asc^−/−^* mice were backcrossed for 9 generations with C57BL/6 mice. *Trif^−/−^* mice were generated on C57BL6 mice and once were backcrossed at Jackson laboratory.

### Reagents

LPS from *E. coli* (InvivoGen, San Diego, CA), recombinant IL-1 receptor antagonist (IL-1RA) (Kineret, Amgen), recombinant IL-1β (eBioscience, San Diego, CA), *N*-Acetyl-L-leucyl-L-leucyl-L-methional (Tocris Bioscience, Ellisville, MO), Mito tracker green (Invitrogen, Carlsbad, CA), adenosine 5″-triphosphate, chloramphenicol, cytochalasin D, staurosporine, and peptidoglycan from *S. aureus* and *N*-Acetyl-L-cysteine (Sigma, St. Louis, MO) were purchased commercially.

### Infection and Bacterial Quantification

Lung homogenates from *C. pneumoniae* (CM-1, ATCC, Manassa, VA) infected mice were propagated in HEp2 cells and counted as previously described [7].

### Histopathological analysis

Lungs were fixed in formalin buffer, paraffin-embedded, and hematoxylin and eosin (H&E)-stained sections were scored by a trained pathologist blinded to the genotypes as previously described [7].

### Detection of cytokines

The cytokine concentrations in the BALF, lung homogenates or culture supernatant were determined using by OptiEIA Mouse IL-6 ELISA Set (BD Biosciences, San Jose, CA, USA) and Mouse IFNγ ELISA, Mouse IL-12p40 ELISA, Mouse IL-1β ELISA and Mouse TNFα ELISA (eBioscience). The assays were performed as described in manufacturers’ protocols.

### Measurement of mitochondrial membrane potential (Δψ_m_)

Cells were stained with the cationic dye TMRM (AnaSpec, Fremont, CA, USA) as described in the manufacturer’s protocol. Cells were loaded with 200 nm TMRM for 30 min, washed three times with PBS and fluorescence was measured using a SpectraMaX M2 Microplate Reader (Molecular Devices Corp., Sunnyvale, CA, USA) or by fluorescence microscopy (Nikon Eclipse T2000).

### Measurement of ROS production

Cells were incubated in phenol red-free RPMI1640 medium containing 10 µM 6-carboxy-2',7'-dichlorodihydrofluorescein diacetate (Molecular Probes, Eugene, OR) for 30 min and then infected with *CP*. The loading buffer was removed, washed and fluorescent intensity was measured using a microplate reader.

### Measurement of mitochondrial oxygen consumption

Oxygen consumption rates (OCR) were measured using an XF24 Extracellular Flux Analyzer (Seahorse Bioscience). For the XF24 assay, cells were equilibrated with DMEM lacking bicarbonate at 37°C for 1 hour in an incubator lacking CO_2_. Mixing, waiting, and measurement times were 0.5, 2, and 3 min, respectively (an extra 0.5 min was added after each injection). Oligomycin, blocks phosphorylation of ADP to ATP, thus preventing mitochondrial respiration, and providing a basal level of O_2_ consumption during the assay. FCCP is an uncoupling agent and provide maximal O_2_ consumption under a given condition. Rotenone is a respiration inhibitor that blocks at mitochondrial respiratory complex 1. These were used to show the specificity of the reaction.

### Immunoblot

BMDM were stimulated for indicated time, supernatants were collected and proteins were precipitated by methanol-chloroform extraction, and cell lysates were collected. Immunoblot analysis was done with described antibodies; anti mouse caspase-1 p10 (sc-514; Santa Cruz Biotechnology), anti-mouse IL-1β (AF-401-NA; R&D Systems), anti-GAPDH (6C5; Santa Cruz Biotechnology).

### Tissue damage scoring

Tissue damage was assigned as arbitrary score of 0 (normal  =  no inflammation), 1 (minimal  =  perivascular, peribronchial, or patchy interstitial inflammation involving less than 10% of lung volume), 2 (mild  =  perivascular, peribronchial, or patchy interstitial inflammation involving 10–20% of lung volume), 3 (moderate  =  perivascular, peribronchial, patchy interstitial, or diffuse inflammation involving 20–50% of lung volume), and 4 (severe  =  diffuse inflammation involving more than 50% of lung volume).

### Flow cytometric analysis

Isolated single cells were stained with anti-F4/80 mAb (clone BM8), anti-CD11c mAb (clone HL3). For intracellular iNOS staining, cells were permeabilized using Cytofix/Cytoperm kit (BD Biosciences) and stained with conjugated anti-mouse iNOS mAb (clone 6/iNOS/NOS Type II, BD Bioscienses). The lymphocytic makeup of the lungs after infection was analyzed by flow cytometry of lung homogenates. Briefly, lymphocytes were isolated by digesting the lung tissue at 37°C for 1 h in HANKS buffer containing 100 µg/ml Blendzyme (Roche Diagnostics, Indianapolis, IN, USA) and 50 units/ml DNase I (Roche Diagnostics) and filtering through a 70 µm cell strainer (BD Biosciences). Erythrocytes were depleted by lysis buffer before staining. Isolated single cells were stained with following specific mAbs: CD16/32 (clone 93), Ly6G (clone 1A8), CD11b (clone M1/70), F4/80 (clone BM8), CD11c (clone HL3), CD45 (clone 30-F11), CD4 (clone RM4-5), CD8 (clone 53-6.7), NK1.1 (clone PK136) and B220 (clone RA3-6B2) purchased from eBioscience as direct conjugates to either FITC, PE or PECy5. Cells were identified based on expression of following antigens: pulmonary macrophages (F4/80+ and CD11c+), DC (F4/80− and CD11c+), Neutrophils (Ly6G+ and CD11b+), T cells (CD3+), NK cells (NK1.1+), B cells (B220+ and CD19+). For intracellular *Chlamydia* staining, cells were permeabilized using Cytofix/Cytoperm kit (BD Biosciences) and stained with FITC-conjugated anti-*Chlamydia* LPS mAb (Accurate Chemical and Scientific Corporation, Westbury, NY, USA). Flow cytometric analysis was performed using a CyAn™ flow cytometer (Beckman Coulter) and the data was analyzed using Summit (Dako, Carpinteria, CA, USA) software.

### Preparation of bone marrow–derived macrophages (BMDM)

Femora and tibiae of mice were aspirated with RPMI1640 media. Bone marrow cells were cultured in RPMI1640 medium containing 10% FBS and 15% L929 cell conditioned medium. BMDM were harvested at day 7 and infected with *CP* by centrifugation at 500×g for 30 min.

### Statistics

Data are reported as mean values±S.D. Statistical significance was evaluated by Student’s t test. In the case of survival study, statistical significance was evaluated by Fisher’s exact test. For multiple comparison test, statistical significance was evaluated by one way ANOVA with Tukey’s post-hoc test.

## Supporting Information

Figure S1 **Control staining of intracellular** ***Chlamydia*** **in lung cells (** **Figure 2A** **).** FITC-conjugated mouse IgG1 was used as isotype control.
(TIF)

Figure S2 **Live** ***CP*** **but not UVCP, induces inflammasome in macrophages.** Western blot analysis of IL-1β and caspase-1 in cell lysates and supernatants from BMDC treated with *CP*, UVCP for 24 h and LPS (10 ng/ml) or LPS+ATP (5 mM) for 8+2 hrs (respectively).
(TIF)

Figure S3 **Casp1 deficiency does not affect macrophage phagocytic activity or** ***C. pneumoniae*** **infectivity.** (A) *Casp1^−/−^* macrophages are as effective as WT macrophages in internalizing *CP*. BMDMs were infected with labeled *CP* (solid line histogram, MOI 2.5, 5, 10, and 20) or vehicle control (gray-filled histogram). The mean fluorescence intensity (MFI) and percentage of labeled *C. pneumoniae* internalized cells are indicated. (B) WT and *Casp1^−/−^* BMDMs were infected with *CP* (MOI 10). Cell lysates were harvested at indicated time points and viable bacteria were quantified by infecting HEp2 cells followed by inclusion staining and counting.
(TIF)

Figure S4 **Induced Pro-IL-1β is downregulated without NLPR3 stimuli.** BMDM were exposed to UVCP (MOI 10) or treated with LPS (1 ìg/ml) for the indicated times. Immunoblotting was used to analyze intracellular pro-IL-1β.
(TIF)

Figure S5 **Chloramphenicol prevents** ***C. pneumoniae*** **inclusion formation dose dependently.** (A) Representative images of *CP* inclusion formation (green) with increasing doses of chloramphenicol in Hep2 cells. Hep2 cells were infected with *CP* (MOI 5). After entry into the cells, the media was supplemented with different doses of chloramphenicol. *CP* inclusions were observed 68 hours after infection using the Pathfinder Chlamydia culture testing system (Biorad, CA). (B) Number and size of *CP* inclusions with increasing doses of chloramphenicol in Hep2 cells.
(TIF)
