# Supplementary figures and images for: Caspase-1 Dependent IL-1β Secretion Is Critical for Host Defense in a Mouse Model of Chlamydia pneumoniae Lung Infection
Source: PLoS One. 2011 Jun 23;6(6):e21477. doi: 10.1371/journal.pone.0021477 (PMC3121765; doi:10.1371/journal.pone.0021477)

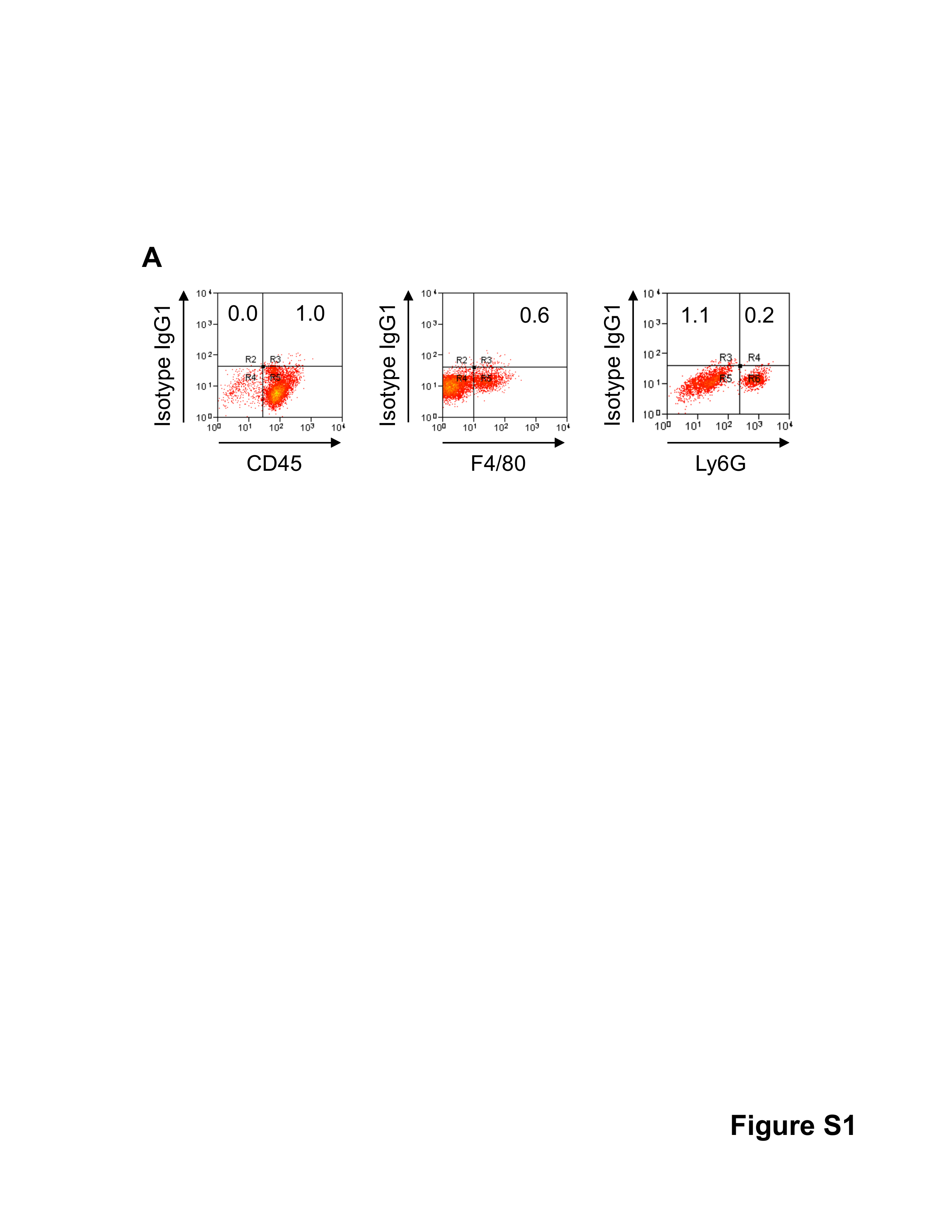

Supplement: Figure S1 — Control staining of intracellular Chlamydia in lung cells ( Figure 2A ). FITC-conjugated mouse IgG1 was used as isotype control. (TIF) [file pone.0021477.s001.tif]

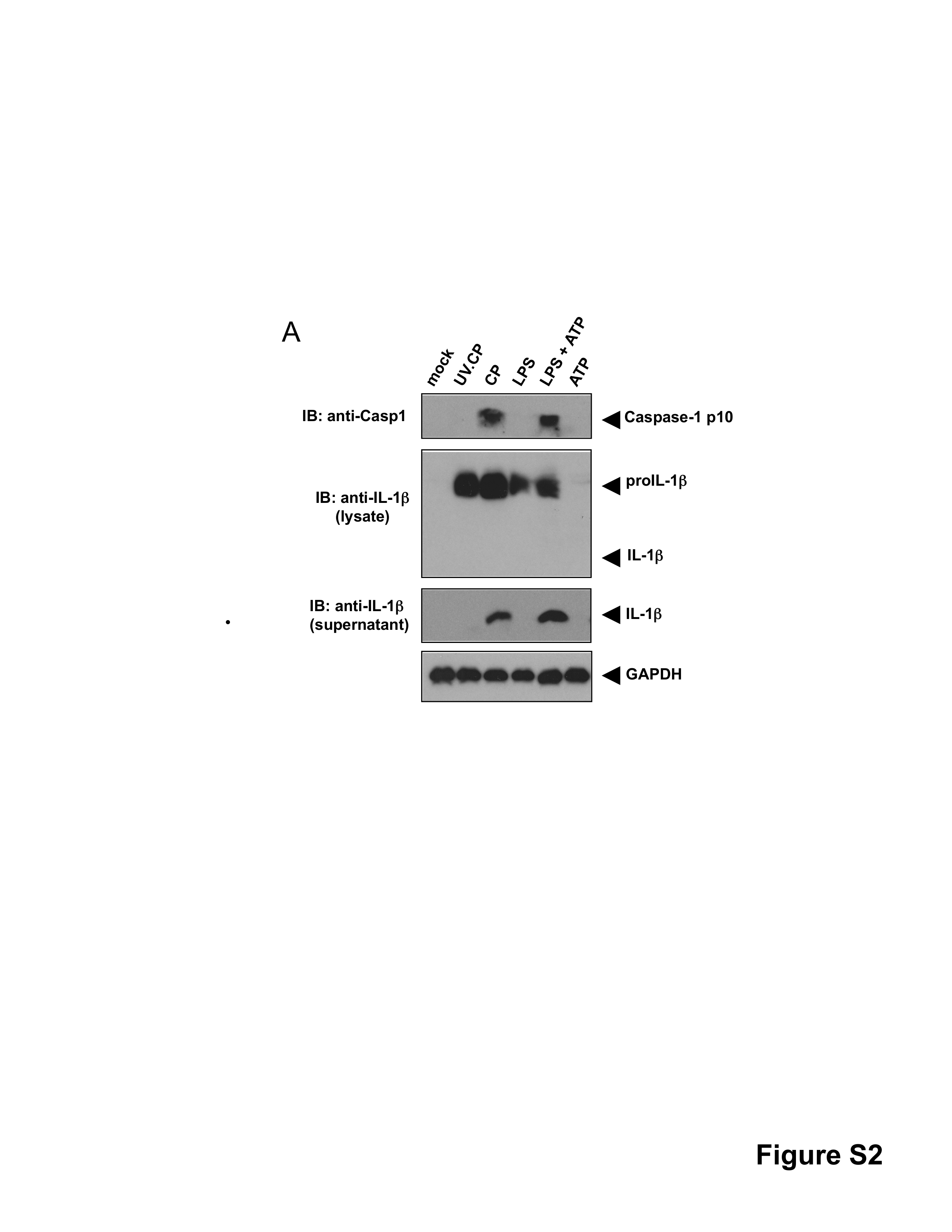

Supplement: Figure S2 — Live CP but not UVCP, induces inflammasome in macrophages. Western blot analysis of IL-1β and caspase-1 in cell lysates and supernatants from BMDC treated with CP, UVCP for 24 h and LPS (10 ng/ml) or LPS+ATP (5 mM) for 8+2 hrs (respectively). (TIF) [file pone.0021477.s002.tif]

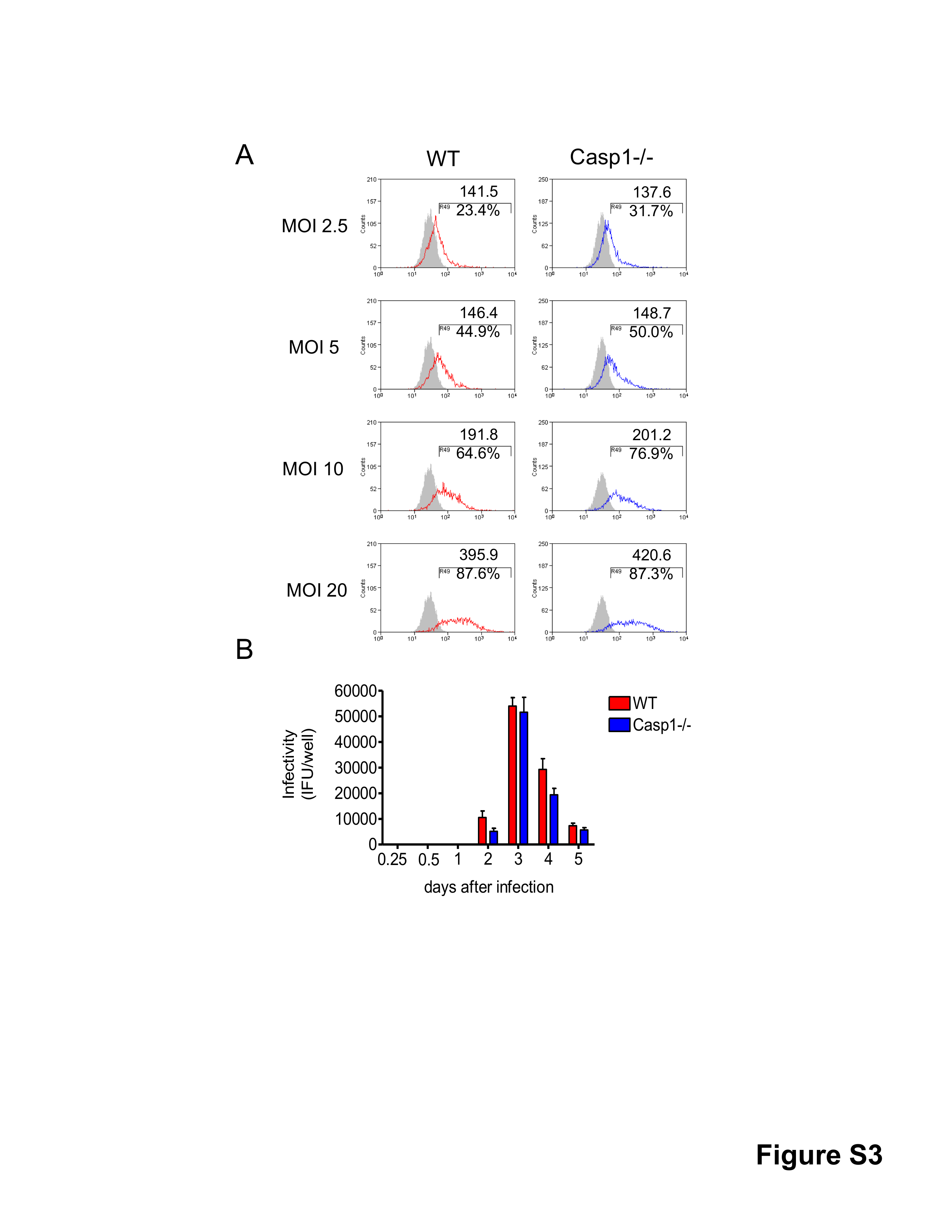

Supplement: Figure S3 — Casp1 deficiency does not affect macrophage phagocytic activity or C. pneumoniae infectivity. (A) Casp1−/− macrophages are as effective as WT macrophages in internalizing CP. BMDMs were infected with labeled CP (solid line histogram, MOI 2.5, 5, 10, and 20) or vehicle control (gray-filled histogram). The mean fluorescence intensity (MFI) and percentage of labeled C. pneumoniae internalized cells are indicated. (B) WT and Casp1−/− BMDMs were infected with CP (MOI 10). Cell lysates were harvested at indicated time points and viable bacteria were quantified by infecting HEp2 cells followed by inclusion staining and counting. (TIF) [file pone.0021477.s003.tif]

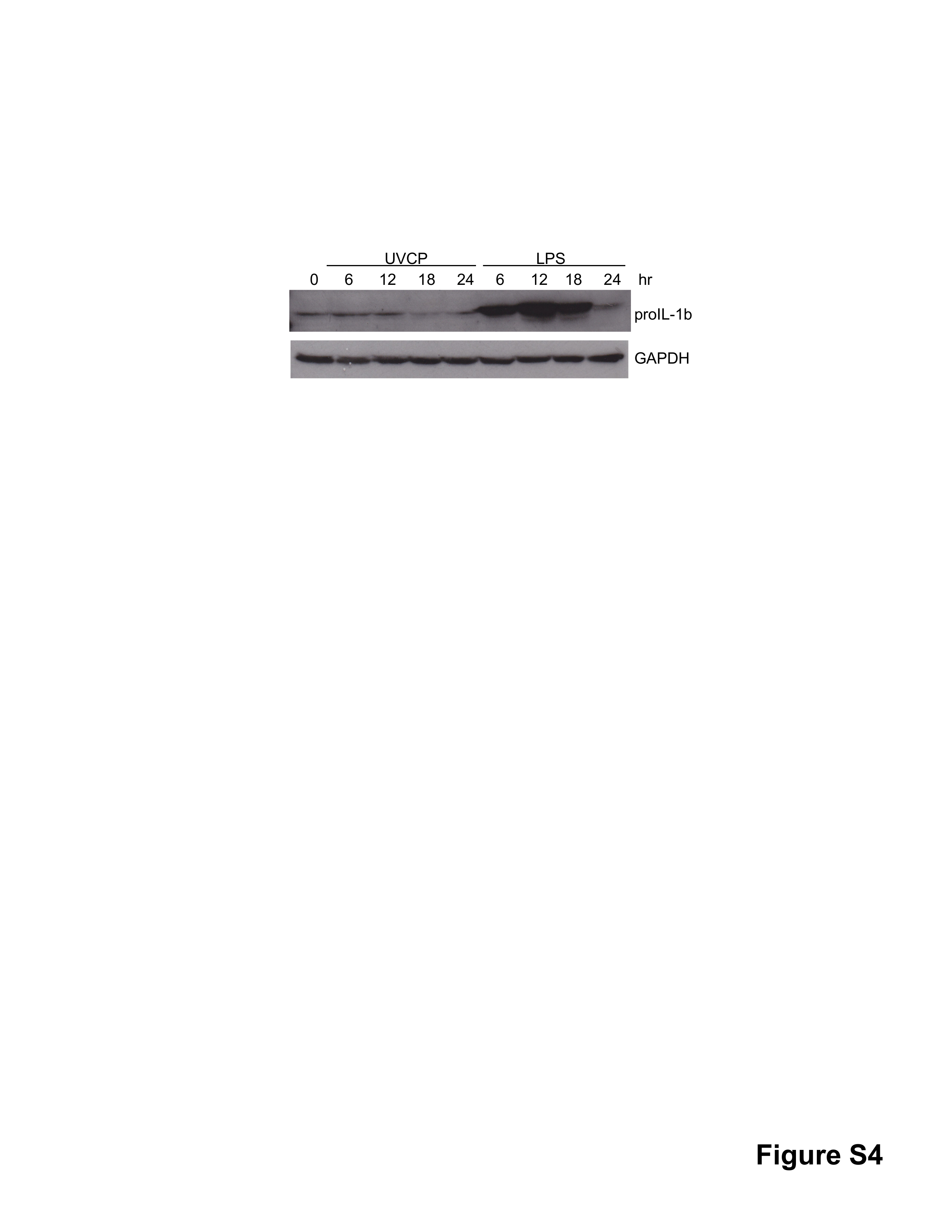

Supplement: Figure S4 — Induced Pro-IL-1β is downregulated without NLPR3 stimuli. BMDM were exposed to UVCP (MOI 10) or treated with LPS (1 ìg/ml) for the indicated times. Immunoblotting was used to analyze intracellular pro-IL-1β. (TIF) [file pone.0021477.s004.tif]

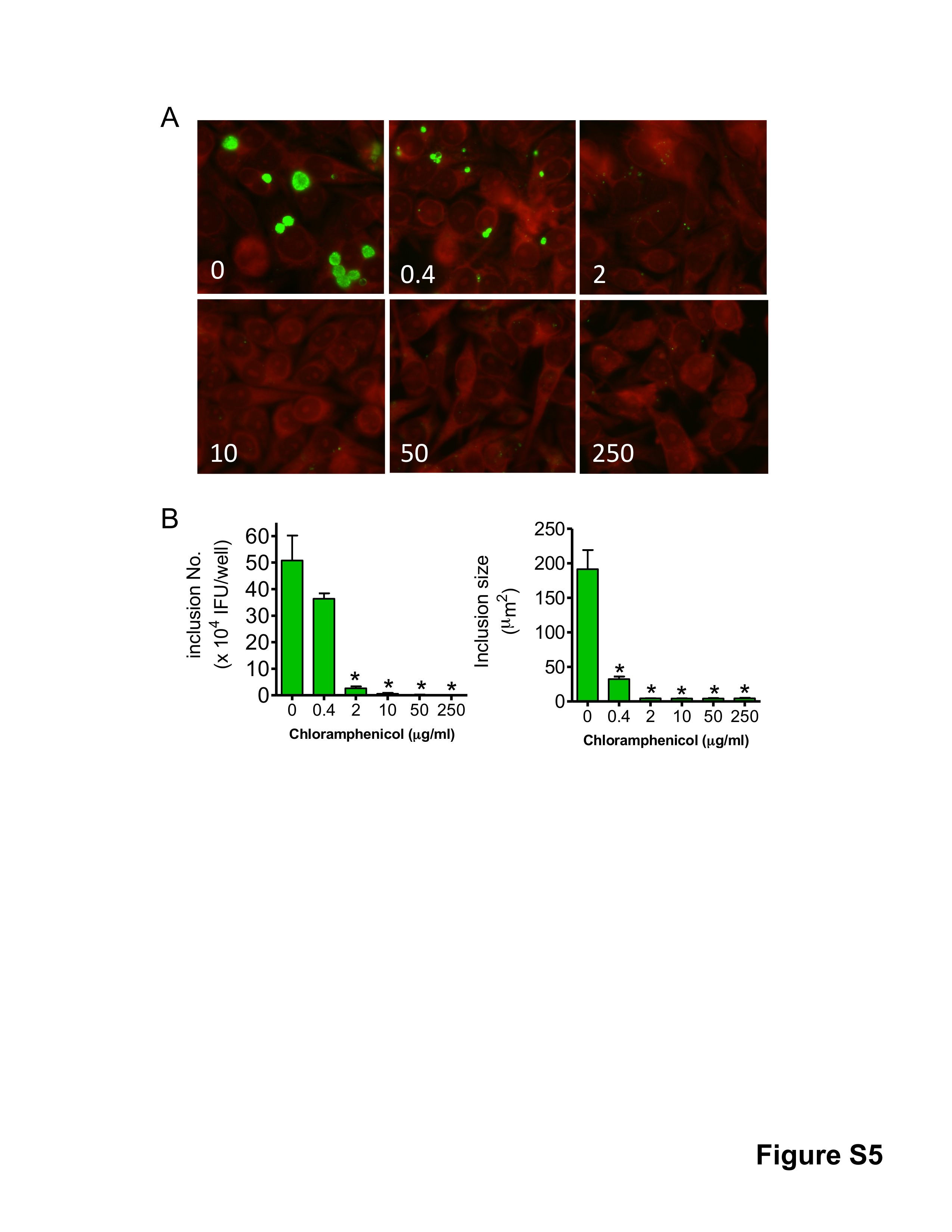

Supplement: Figure S5 — Chloramphenicol prevents C. pneumoniae inclusion formation dose dependently. (A) Representative images of CP inclusion formation (green) with increasing doses of chloramphenicol in Hep2 cells. Hep2 cells were infected with CP (MOI 5). After entry into the cells, the media was supplemented with different doses of chloramphenicol. CP inclusions were observed 68 hours after infection using the Pathfinder Chlamydia culture testing system (Biorad, CA). (B) Number and size of CP inclusions with increasing doses of chloramphenicol in Hep2 cells. (TIF) [file pone.0021477.s005.tif]
